# Small Bowel Obstruction Secondary to Wild Banana Seed Ingestion

**DOI:** 10.4269/ajtmh.19-0586

**Published:** 2019-12

**Authors:** Divya K. Natarajan, Phonexay Homthavong, Indi Trehan

**Affiliations:** 1 Department of Pediatrics, Washington University in St. Louis, St. Louis, Missouri;; 2 Lao Friends Hospital for Children, Luang Prabang, Lao PDR;; 3 Luang Prabang Provincial Hospital, Luang Prabang, Lao PDR

**Figure 1.**
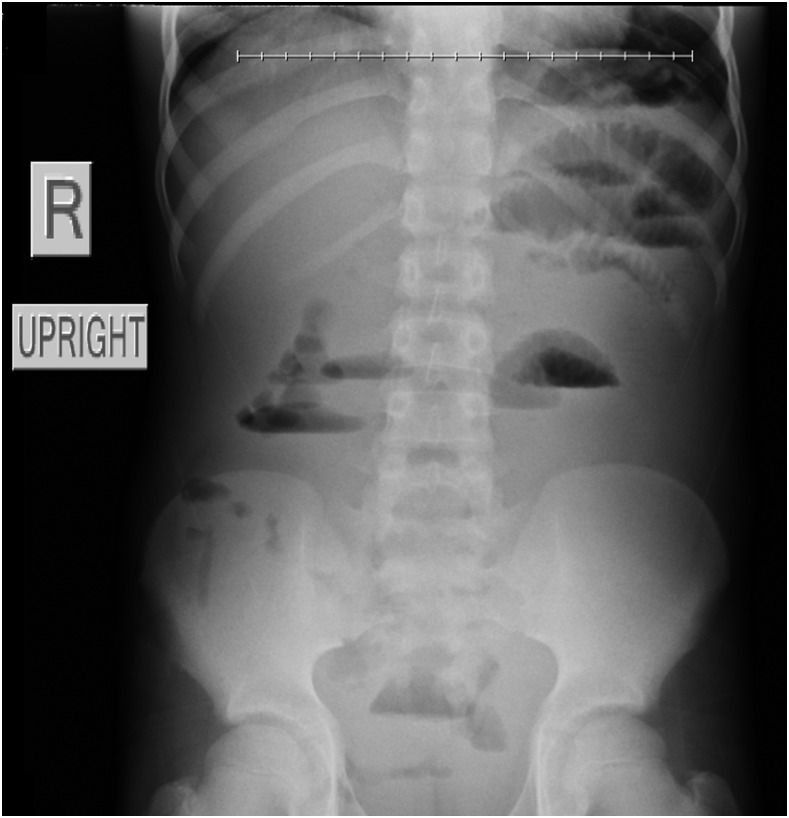
Abdominal X-ray demonstrating small bowel obstruction.

**Figure 2.**
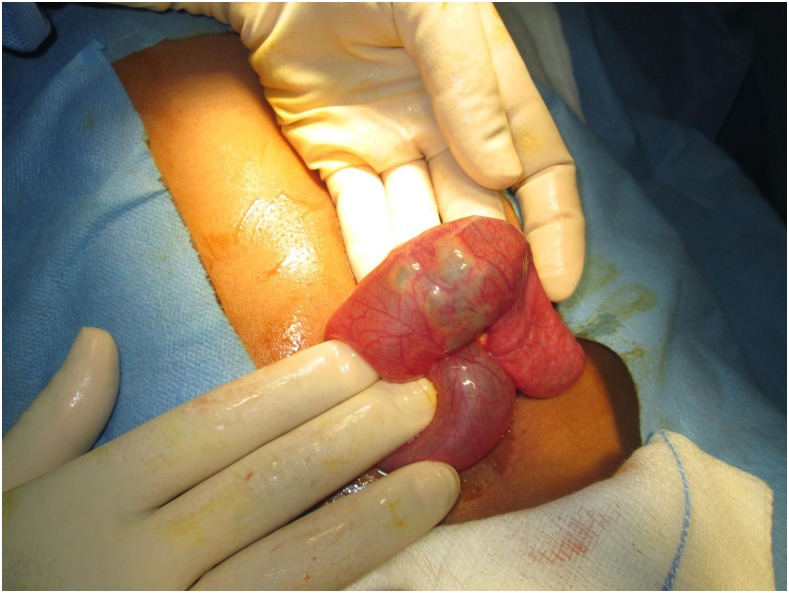
Intraoperative finding of bezoar causing bowel obstruction. Individual banana seeds are visible through the intestinal lumen. This figure appears in color at www.ajtmh.org.

**Figure 3.**
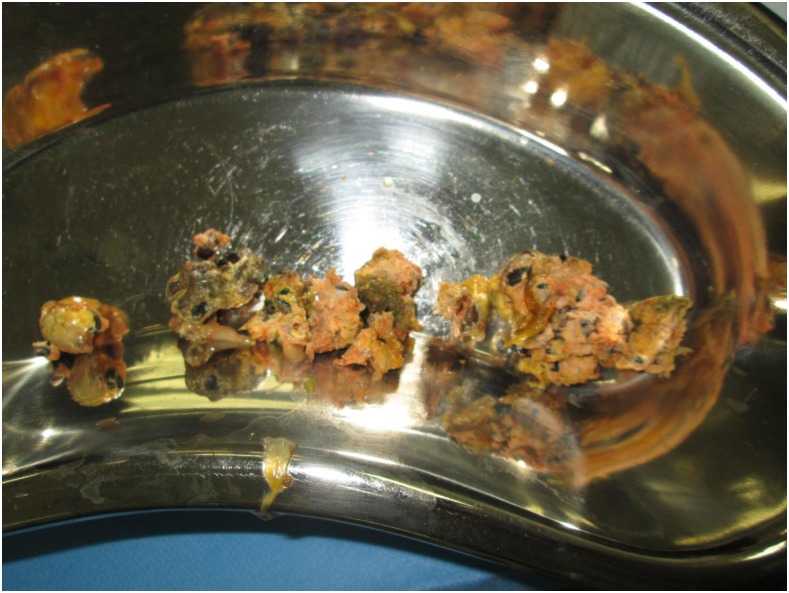
Phytobezoar specimens extracted from the bowel. This figure appears in color at www.ajtmh.org.

A previously healthy 13-year-old boy from a rural village in northern Laos presented with progressive abdominal pain, constipation, emesis, and marked abdominal distention. Radiographs showed a small bowel obstruction which did not resolve with conservative management (Figure 1). At laparotomy, a bezoar was identified as the cause of his bowel obstruction (Figure 2). Resection of the mass identified a phytobezoar of banana seeds (Figure 3). The patient tolerated surgery well and had an unremarkable postoperative course.

Further history obtained postoperatively revealed that before presentation, the patient was hungry and foraging for food for several days as his family was without means and reliable access to food. He chanced on a wild banana tree and indulged in its fruits.

**Figure 4.**
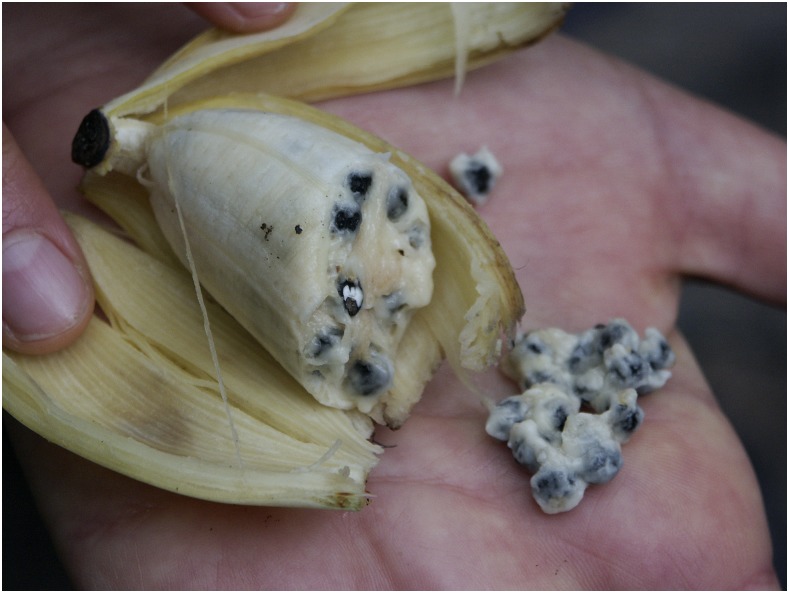
Wild banana plant *Musa balbisiana* native to Southeast Asia depicting large seeds capable of causing intestinal obstruction through formation of phytobezoar. (Image courtesy of Scott Zona.) This figure appears in color at www.ajtmh.org.

The offending fruit, *Musa balbisiana*, is a wild banana species native to Southeast Asia, spanning from India to Papua New Guinea (Figure 4). Ingestion of the fruit seeds is known to cause intestinal complications, including constipation, appendicitis, and small bowel obstruction, most commonly in rural, impoverished populations because of limited access to safe nutrition.^1–4^ Despite local wisdom to avoid these dangerous fruits and multiple reports of wild banana ingestion–related bowel obstruction, cases like this demonstrate the impact that food insecurity and starvation can have on impoverished populations.
